# Hospital Mortality – a neglected but rich source of information supporting the transition to higher quality health systems in low and middle income countries

**DOI:** 10.1186/s12916-018-1024-8

**Published:** 2018-03-01

**Authors:** Mike English, Paul Mwaniki, Thomas Julius, Mercy Chepkirui, David Gathara, Paul O. Ouma, Peter Cherutich, Emelda A. Okiro, Robert W. Snow

**Affiliations:** 1 KEMRI-Wellcome Trust Research Programme, P.O. Box 43640, Nairobi, 00100 Kenya; 2 Centre for Tropical Medicine and Global Health, Nuffield Department of Medicine, University of Oxford, Oxford, UK; 3 Department of Preventive and Promotive Health, Ministry of Health, Nairobi, Kenya

## Abstract

**Background:**

There is increasing focus on the strength of primary health care systems in low and middle-income countries (LMIC). There are important roles for higher quality district hospital care within these systems. These hospitals are also sources of information of considerable importance to health systems, but this role, as with the wider roles of district hospitals, has been neglected.

**Key messages:**

As we make efforts to develop higher quality health systems in LMIC we highlight the critical importance of district hospitals focusing here on how data on hospital mortality offers value: i) in understanding disease burden; ii) as part of surveillance and impact monitoring; iii) as an entry point to exploring system failures; and iv) as a lens to examine variability in health system performance and possibly as a measure of health system quality in its own right. However, attention needs paying to improving data quality by addressing reporting gaps and cause of death reporting. Ideally enabling the collection of basic, standardised patient level data might support at least simple case-mix and case-severity adjustment helping us understand variation. Better mortality data could support impact evaluation, benchmarking, exploration of links between health system inputs and outcomes and critical scrutiny of geographic variation in quality and outcomes of care. Improved hospital information is a neglected but broadly valuable public good.

**Conclusion:**

Accurate, complete and timely hospital mortality reporting is a key attribute of a functioning health system. It can support countries’ efforts to transition to higher quality health systems in LMIC enabling national and local advocacy, accountability and action.

## Background

*“An urgent appeal for adopting…some uniform system of publishing the statistical records of hospitals. If they could be obtained…they would show subscribers how their money was being spent, what amount of good was really being done with it, or whether the money was doing mischief rather than good.” (Attributed to Florence Nightingale, 1863)*

The desire for better health statistics is not new. With 2030 defined as the next major global health horizon the majority of targets and indicators related to the 3rd Sustainable Development Goal (SDG3, ‘Ensure healthy lives and promote well-being for all at all ages’) require an ability to measure population health status [1]. We expect a national health information system (HIS) to furnish such measurement. Indeed, Abou-Zahr et al. suggest the HIS should address the following domains [2]: health determinants (socioeconomic, environmental, behavioural and genetic factors); inputs to the health system including health infrastructure, facilities and human and financial resources; health outcomes (mortality, morbidity, disability, well-being, disease outbreaks and health status); and inequities in determinants, coverage and use of services, and outcomes. Here we focus on one specific level of the primary health care system and value of the information it can yield.

There is no universally agreed definition of a hospital [3]. For the purposes of this report we are most concerned with facilities that should provide inpatient and ambulatory care to populations at district or regional levels (hereafter referred to just as district hospitals). In larger district hospitals (likely to have a total of 80 inpatient beds and often many more) norms and standards in low and middle-income countries (LMIC) often aspire to have services led by at least one obstetrician, paediatrician, physician and surgeon supported by basic laboratory and imaging diagnostic resources. Such facilities are often also centres providing experiential training to multiple health worker cadres. The Alma Ata Declaration clearly states that these facilities are a critical part of the primary health care system [4]. We argue they could and should offer valuable insights on health system needs and performance. Yet their role has largely been ignored for many years. As the SDGs focus attention on reinvigorating primary health care efforts must include understanding and strengthening district hospitals as part of these systems.

## Our argument

It is a fundamental attribute of a health system that it can report hospital mortality accurately. We argue that such reporting and its quality is neglected despite seeming a much more achievable measurement goal than many others. While there is a need for much more research on how future district hospitals can support primary health care goals of universal coverage we focus specifically here on the value of better hospital mortality data as an immediate concern: i) in understanding disease burden, ii) as part of surveillance and impact monitoring, iii) as an entry point to exploring system failures, and iv) as a possible measure of health system quality in its own right. We present these arguments below illustrating them with data from the Kenyan health system where appropriate.

### Hospital mortality as a window on burden of disease

A decade ago it was suggested that absence of reliable data for births, deaths, and causes of death contributes to a ‘scandal of invisibility, which renders most of the world’s poor as unseen, uncountable, and hence uncounted’ [5]. Death registration is worst in African and Asian regions [6]. Thus, we continue to rely on sparse data from a small number of population-based studies that employ verbal autopsy interviews with bereaved relatives about symptoms and signs to attribute these to a specific cause. These data are then modelled and interpolated in time and space to provide an estimate of cause-specific mortality for every year since 1980 in every country [7]. These estimates have been important for disease burden estimation and global priority setting providing at least some estimates for the number of deaths attributable to specific causes in many low-income countries. Yet, they are often based on limited or no suitable local data and have wide confidence intervals. This seems inappropriate as we approach the third decade of the twenty-first century [8, 9].

**Fig. 1:**
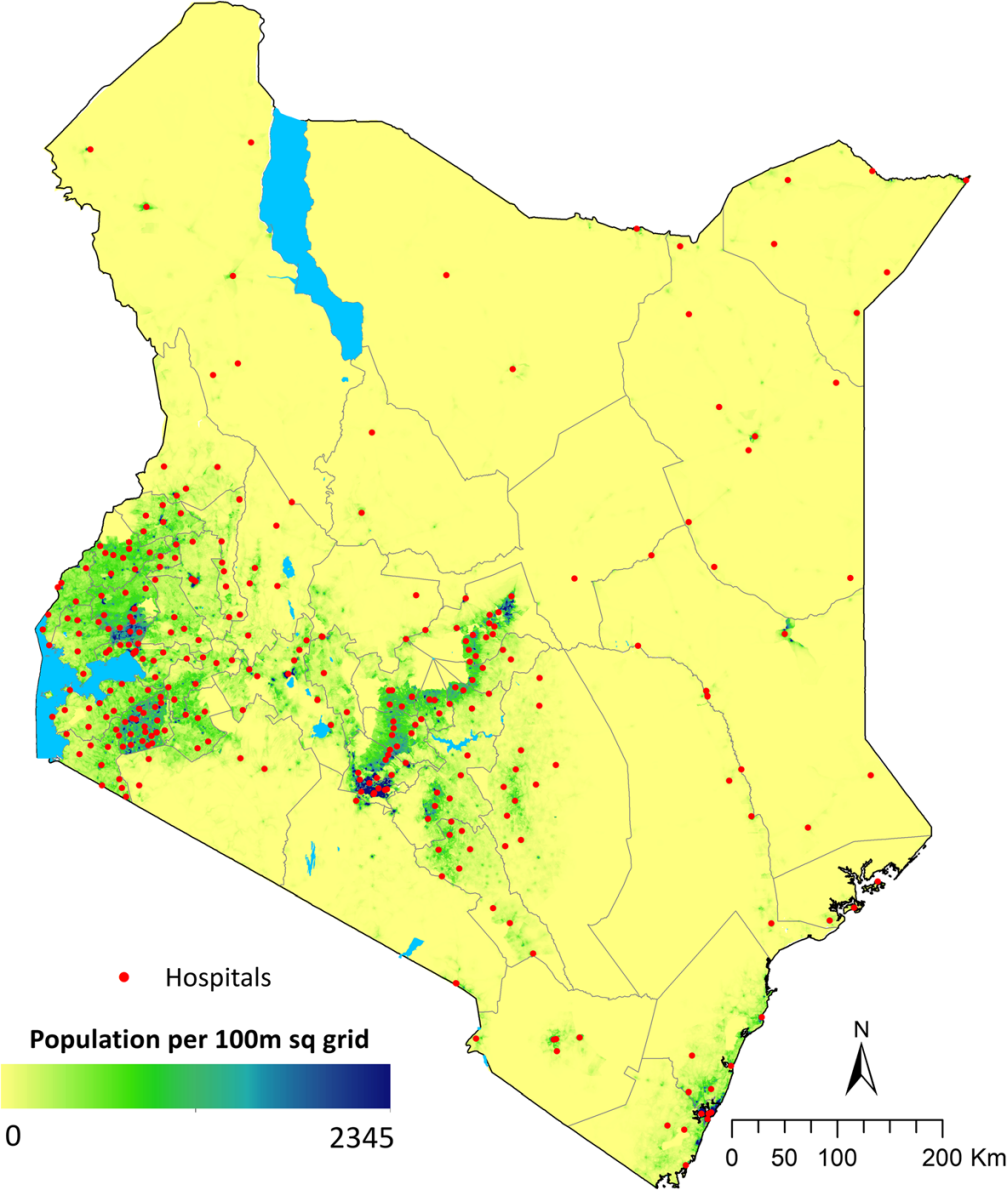
Distribution of hospitals and population density in Kenya. This figure shows the distribution of hospitals in Kenya in relation to population density suggesting they could offer insights on cause of death in diverse geographic settings spanning high and very low density populations in Kenya. Of note Kenya has demographic surveillance sites conducting verbal autopsy in 5 locations none of which are in areas of low population density

Better reporting of cause-specific hospital mortality is a major missed opportunity to dramatically increase the availability of cause of death data. Although we need to recognised the potential bias in these data, related to the proportion of the population with access to hospital care, cause of death reporting from hospitals where individual cases can be reviewed by trained health-workers with access to diagnostic procedures should be more accurate and timely than data derived from interview responses with bereaved family members – and of course professionally determined cause of death data are the norm in in high income countries. Hospital data are also available for many more sites and time points across LMIC than those from population-based, verbal autopsy surveys (and see Fig. 1). We should be using such data in national policy and planning and to triangulate, calibrate or pressure test model predictions of the frequency or trends in cause-specific mortality. High quality hospital cause of death data should also be allied with improved routine civil registration and vital status. Together these would make a major contribution to our local understanding of disease burden and its variation and be an important advocacy tool helping to prioritise resource allocation. Improved hospital data will become especially important as many LMIC enter the epidemiologic transition providing opportunities for extended time series analyses that would be highly informative.

**Fig. 2:**
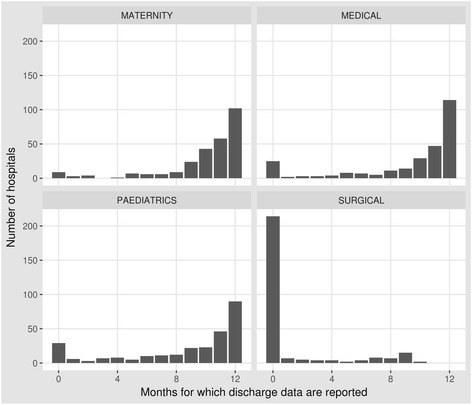
Reporting rates from 272 hospitals for discharges from major service units in Kenya. This bar chart demonstrates the distribution of the number of months for which there are discharge data from four major inpatient service units (maternity, paediatric wards, medical wards and surgical wards) in 272 Kenyan hospitals for the year December 2015 to November 2016

Taking advantage of what should be readily accessible cause of death information will require us to make efforts to promote its accuracy. This will require better training for health workers who document diagnoses and cause of death in most routine settings [10]. Inadequate investments in information systems generally and in this area specifically are one reason why external assessments of the accuracy of hospital cause of death data in LMIC suggest it can be poor [11, 12] and incomplete [13]. Illustrating this we display in Box 1 the frequency of missing data on hospital mortality from 272 public hospitals in Kenya [14], with data missing for an entire 12 months on surgical admissions and outcomes in over 200 hospitals. Yet addressing these challenges is arguably more tractable in the long-run than increasing coverage with sophisticated verbal autopsy approaches to determine cause of death in dedicated demographic surveillance systems. Enabling countries to generate quality health information at national and sub-national levels in the same way that high income countries do is also clearly preferable to a future that continues to rely on modelling, and the uncertain estimates it produces, to understand local disease burdens (Fig. 2).

### Hospital mortality – value in surveillance and impact evaluation

Tracking specific hospital mortality events is an important element of Integrated Disease Surveillance and Response (IDSR) [15] with hospitals in many LMIC expected to report both the frequency of priority illnesses and their outcome. Thus, an increase in deaths associated with characteristic features of meningitis, Cholera or Ebola may signal the onset of an outbreak or epidemic of national (or even international) importance [16, 17]. Such tracking may extend to syndromic surveillance. For example, for severe acute respiratory syndrome (SARS) a rapid rise in cases and case fatality may be an important signal. Good hospital mortality data may also be key to monitoring the quality and coverage of routine health programs. Deaths due to illness such as neonatal tetanus may signal the failure of vaccination efforts, deaths attributable to malaria and TB to deficiencies in specific programmes [18, 19]. Both could signal important regional differences [20]. Increasingly mortality data may also be a key part of national disease registries (eg. for trauma or cancers) that inform long-term planning and monitoring of outcomes [21].

For specific conditions hospital mortality data has been used to evaluate the quality of service provision too. For example, to track efforts to improve outcomes after acute myocardial infarction in high income settings [22] while perioperative mortality is proposed as a key indicator of quality of surgical services in LMIC [23]. Recent studies also suggest that good quality data on hospital mortality may support evaluation of large-scale quality improvement (QI) programmes in LMIC. The ‘Project Fives Alive!’ in Ghana employed a strategy to improve maternal and child health outcomes using a QI methodology to recognize barriers to care-seeking and care provision at the facility level and then to identify, test and implement simple and low-cost local solutions that addressed these barriers. In the impact evaluation, the intensity of the QI activity was associated with a reduction in hospital mortality for those aged 0 – 59 months. However, the strength of these conclusions was undermined by the fact that 43% of data on hospital outcomes were missing [24, 25]. Most recently a multilateral partnership supporting work in nine LMIC was initiated with an aim of reducing in-facility maternal and neonatal mortality by 50% [26]. The health impact of such investments will remain a matter of conjecture without accurate mortality data from multiple facilities over prolonged periods. Greater coordination of effort and investment by all parties to improve hospital mortality data that can serve the needs of multiple programmes would also help create data as a broader public good.

### Hospital mortality and system failure

Striking headlines suggest up to one third of deaths in the USA are attributable to medical errors although this is probably a gross overestimate [27, 28]. Despite differences of opinion on headline figures there is agreement that there is considerable scope to improve quality and safety even in high income settings [29]. But, is mortality a good measure of the quality of a hospital’s care? This question is much debated and we return to it below. However, it is now widely felt that appropriately structured and detailed analyses of individual inpatient deaths that identify deficiencies and errors in care can contribute to improvements in quality and safety of health systems [30, 31]. Examination of maternal deaths has been used for over 50 years in higher income countries and has profoundly influenced policy and practice at multiple levels of the health system [32]. This approach is strongly promoted in LMIC through programmes focused on maternal (and more recently perinatal) death reviews [33]. However, implementation remains patchy and impacts uncertain. In South Africa systems have been developed to examine deaths of children in hospitals and identify the nature and prevalence of factors that could be modified to reduce mortality [34]. Now operating at considerable scale reports suggest information aggregated across multiple facilities is influencing policy and resource allocation aimed at improving services equitably [34]. Case based investigation of hospital mortality that spans examination of pathways to care may thus be a form of ‘system diagnostic’ that is directly useful for local quality improvement and useful well beyond the hospital to support advocacy and accountability [35].

**Fig. 3:**
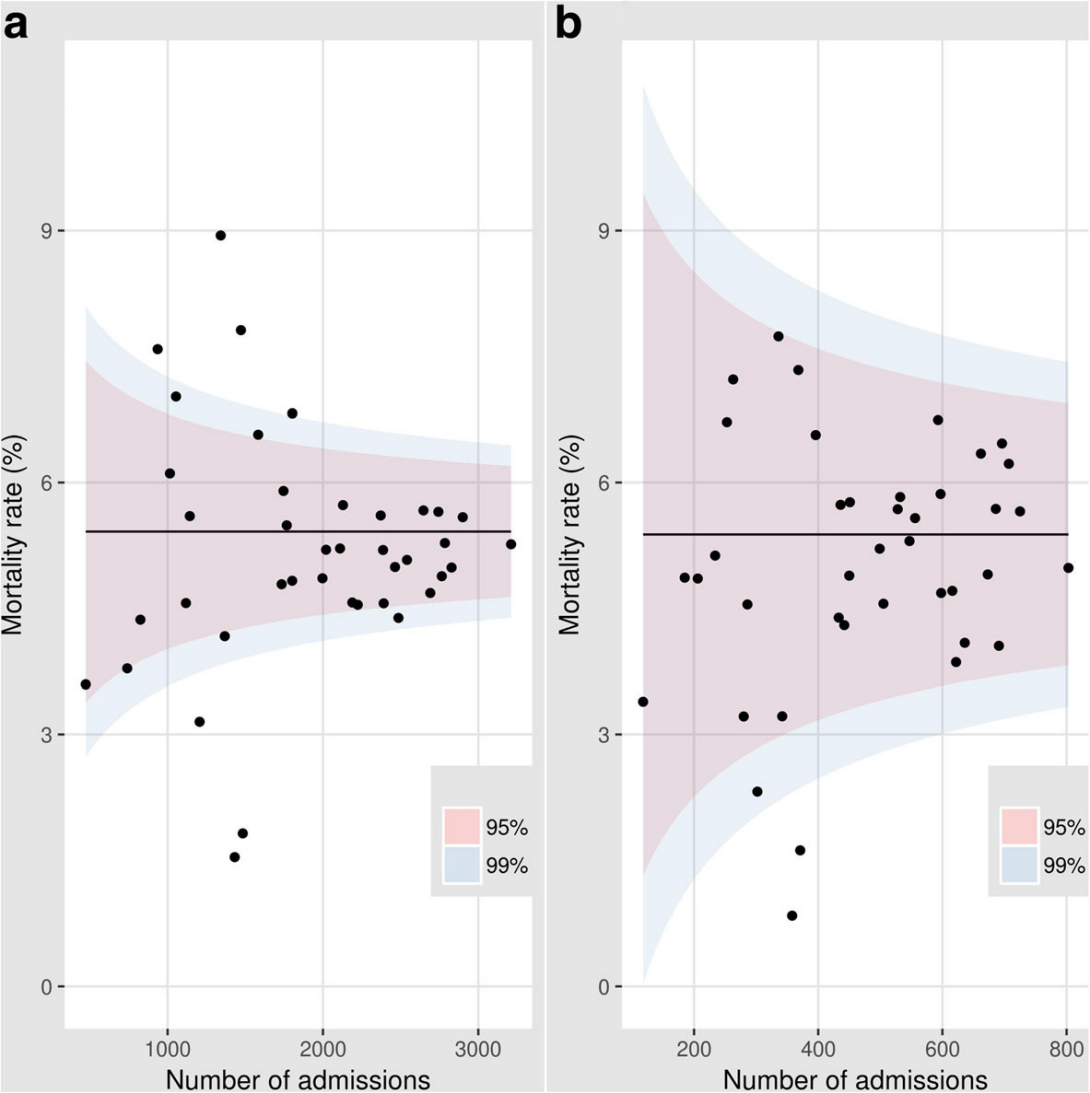
Using funnel plots to explore variability in hospital outcomes. Mortality rates (Y axis) are plotted against the annual number of eligible cases (X axis) for 40 hospitals with a horizontal line indicating the sample mean derived from the 40 observations and the inner and outer shaded areas indicating the 95% and 99% ranges respectively. In panel a (left) we vary the number of eligible cases from 0 to 4000 representing the range seen in our empirical data. In panel b (right) we randomly eliminate cases to reduce the sample size by 75% in each hospital to illustrate the effect on the 95% and 99% ranges. The reduction in case numbers available, as seen in the right panel, illustrates its effect on the potential for identification of outlying values and indicates that such analyses are likely to be most suitable for exploring variation across larger facilities or at sub-national regional levels

Aggregate or service specific hospital mortality data may also raise flags over system failure. Identification of persistently high adult inpatient mortality in one United Kingdom hospital and of high mortality following paediatric cardiac surgery in another triggered national enquiries that reported major failings in clinical and management processes that had implications for the whole health sector [36]. As LMIC introduce more specialist (and often expensive) forms of intervention delivered through its hospitals attention should be paid to accurate and transparent reporting of mortality. For example, Kenya has recently made major investments in equipment to support the provision of intensive care and renal replacement therapy. Careful scrutiny of the outcomes of such services should be an important part of examining the value of these investments while also seeking to identify where systems need to be strengthened [37]. While it remains challenging to compare hospitals’ mortality rates fairly (see below) even basic data may begin to prompt engagement of policy makers, managers and practitioners in a process of exploring reasons for variability helping direct action to improve services [38–40]. However, it is clearly important that there are sufficient, high quality data to support such a process of inquiry [41, 42]. To illustrate how relatively simple visual representations of hospital mortality data might engage multiple stakeholders to discuss and explore the reasons for variability we use empiric and simulated data on inpatient child mortality from Kenyan hospitals in Box 2 (Fig. 3).

### Hospital mortality as a measure of the quality of care

Can comparison of mortality rates across hospitals provide information on the quality of care provided? This question remains highly contentious. Clearly mortality may be affected by the mix of cases included in the population in which mortality is measured (case-mix) and a range of other, potential risk factors such as a patient’s age or stage of disease (case-severity) [41–43]. To correct for these factors that vary at the individual patient level some high-income countries employ sophisticated adjustment approaches. A number of hospital mortality measures are used in high-income countries and are recommended by Organisation for Economic Co-operation and Development (OECD) and national governments as part of portfolios of quality indicators and there is little doubt such measures (and the debate around them) have directed nations’ attention to improving quality and outcomes. Examples include the UK’s Summary Hospital Mortality Index or Medicare’s more disease specific acute myocardial infarction 30-day mortality rate that are both used as indicators of hospital performance.

So, could specific hospital mortality data be useful in gauging the quality of health care and health systems in LMIC? Two such measures are already part of WHO’s 100 Core Indicators for global tracking and comparison, peri-operative mortality and institutional maternal mortality rates. These, and other measures may be useful for three reasons: a) for benchmarking particularly in conditions where case-specific mortality may be informative, b) for examining the relationship between hospital mortality and variation in health system inputs, and c) for contrasting mortality across hospitals with high workloads in situations where mortality may be sensitive to the quality of care delivered.

#### Benchmarking

Benchmarking against peers (as in Box 2) or a proposed standard may provide useful insights. For example, gestation specific neonatal mortality rates have been used to drive system improvements with the best performers potentially providing an aspirational standard while cross-hospital analyses have informed the organisation of services in high income settings [44, 45]. Another example pertinent to LMIC is that the World Health Organisation suggests that inpatient mortality from complicated severe acute malnutrition in children can be reduced to less than 5% [46]. Where mortality from this condition is much higher it may suggest inadequacies in the overall health system and hospital response to this condition [47] providing the motivation to identify and tackle the reasons for poor outcomes.

#### Examining relationships between specific inputs and outcomes

More broadly, mortality data may be used to examine whether improving health system inputs influences outcomes. In high income settings, for example, variations in workforce capacity have been shown to influence both process measures of quality and hospital mortality [48]. In the case of severe childhood anaemia, an important cause of mortality in many African countries often attributable to malaria, failure to initiate blood transfusion on the day a clinician requests it has been associated with an 80% increase in the odds of hospital death [49]. In Papua New Guinea reductions in hospital mortality were seen after efforts to improve the provision and use of oxygen for childhood pneumonia [50].

#### Inpatient mortality as a possible measure of the overall quality of hospital care

**Fig. 4:**
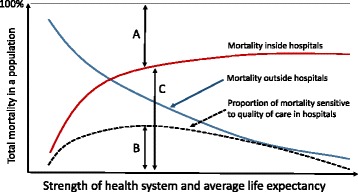
The sensitivity of outcomes of LMIC hospital care to improved quality. This figure seeks to represent hypothetical relationships between the proportion of all mortality (Y Axis) that occurs outside and inside hospitals (blue and red lines respectively) as the strength of a health system and life expectancy increase (X axis). Here we assume that a proportion of all mortality is sensitive to quality of hospital care (dashed line) that first increases as access improves and then decreases as quality improves in parallel with an increase in the strength of a health system. In this simplified model the relationship between the distances represented by A and C is a measure of access that may be particularly important for conditions for which hospital based care might improve outcomes (eg. trauma; acute myocardial infarction; complications of childbirth; preterm birth). If the distance B represents the proportion of mortality that may be averted by better access to higher quality hospital care then in LMIC it is possible that the ratio of B:C (avoidable mortality) is considerably higher than in high income countries (located at the right extreme of the Y axis). With appropriate case-mix and case-severity adjustment mortality may therefore, be a better global metric of quality in LMIC hospitals than it is in high income countries

Above we alluded to the fact that mortality proportions need to be interpreted with great care as variation in the case-mix or illness severity at hospital presentation have a major influence on observed mortality rates even after efforts at statistical adjustment [41, 51, 52]. One key concern is that hospital mortality is a poor overall metric of quality because such a small fraction of deaths are likely to be sensitive to changes in quality of within hospital care [29, 42]. However, two features of LMIC hospitals suggest that examining specific mortality rates may be more informative. Firstly, in some hospital departments only a few serious illnesses may be responsible for the majority of deaths. For example, small numbers of conditions are felt to account for over 60% of paediatric deaths [20]. In well-defined populations case-mix differences may be reduced offering some prospect of effective adjustment [40]. Secondly, although we have only few reports, it seems likely that many more deaths in LMIC hospitals might be sensitive to differences in quality of in-hospital care. Thus improvements in care at the time of delivery resulted in a 15% reduction in maternal mortality in one large study [53] and over 40% of hospital deaths of children in South Africa are thought potentially avoidable [54]. The possible use of hospital mortality data from specific populations as one indicator of the quality of hospital care in LMIC would however, depend on the availability of high quality individual level data from facilities with at least moderately high inpatient workloads (see Box 2). At least in one clinical arena recent research suggests obtaining better quality routine data on individual cases might be possible if simple standardised record forms are introduced [55] and could be facilitated by emerging efforts to implement electronic medical records at scale [56]. In Fig. 4 we present in slightly more detail a hypothetical case for why cause or group specific mortality may be a more sensitive indicator of the quality of hospital care in LMIC than it is in high income countries.

## Conclusion

In an era when even those in rural areas of LMIC have increasing access to digital tools that support their communication and business needs it seems a major paradox that countries are unable to count and characterise deaths in hospitals. Accurate, complete and timely hospital mortality reporting would seem a basic indicator of a quality health system. As access to care expands, better hospital mortality data should offer a granular picture of the burden of disease in a given geography taken together with reporting of all deaths through vital status reporting. It can therefore support advocacy based on population needs and promote equity by highlighting regional variation. More detailed examination of mortality rates, or for selected cases all the events leading up to death, can be important tools to identify system failings. Hospitals themselves, and most importantly their patients, may benefit from benchmarking mortality rates with peers or against aspirational standards to drive local action. In some cases, specific hospital mortality rates may offer a global metric of health care quality in settings where the potential for improvement is large.

The scale of the challenge to improve mortality reporting from at least larger hospitals within LMIC seems, in principle, less daunting than the challenge of implementing the large number of global indicators being discussed as part of efforts to track and improve quality of care. As we seek to strengthen primary health care systems we must not neglect district hospitals as have done in the past. As one first step we believe efforts must be made to improve analysis of hospital mortality data (and suggest specific initial steps in Box 3). These data are a potentially rich source of information supporting the transition to higher quality health systems in LMIC and taking advantage of available technologies can increase the speed with which we can use data for advocacy, accountability and action.

## Box 1 – Hospital mortality reporting, an example from Kenya

In common with 27 countries Kenya utilises the District Health Information System (DHIS) platform as the primary architecture supporting its health information needs. Here we provide some insight into data availability within Kenya’s DHIS system at the level of a county hospital or larger facility. Here a professional health records information officer should lead a team responsible for monthly national reporting using standardised data capture tools. Facilities from all sectors, public, private and not-for-profit, are obliged to report accurate data under the authority of Kenya’s Health Care Bill. Reporting on hospital inpatients in Kenya, including on deaths, should comprise monthly reporting by service unit (eg. maternity, adult medical wards) and by cause using the 10th revision of the International Classification of Disease. In earlier work, we reported that on average 3% of hospital income is used to support information needs and that only half of health records officer positions were filled [13]. We therefore suggest it is underinvestment in information systems as illustrated in panel that is a primary barrier to better reporting of hospital workloads and mortality.

## Box 2 – The use of simple funnel plots to illustrate variability in hospital mortality

Funnel plots are intended to “discourage inappropriate ranking while providing a strong visual indication of divergent performance or special cause variation” [38]. Here, to illustrate their potential use in one LMIC setting we use data collected on all-cause mortality amongst admissions aged 1 to 59 months from 19 county referral hospitals for a single year. We supplement these empiric data with simulated data (derived from the observed data) to create event rates for an additional 21 hypothetical hospitals (creating 40 ‘hospitals’ in total) as funnel plots are a more powerful tool as the number of observation points increases. As each point represents the mortality found in an individual hospital it is possible to identify hospitals that have an apparently high or low mortality (above and below the 95% or 99% range respectively) that are negative or positive ‘deviants’ after taking account of greater uncertainty in mortality proportions derived from smaller populations. Rather than crudely assuming that a high mortality can be equated with a poorly performing hospital (and the converse for low mortality) further exploration of the data are warranted. This may reveal a high mortality hospital receives patients with higher risk of death, for example, that may justify an increase in its resource allocation and further examination of the primary care system with which it is associated to seek explanations.

## Box 3 – Initial steps to improve the production and use of hospital mortality data

1. Conduct and institute regular national audits of mortality reporting from all hospitals, provide feedback to hospitals on their reporting and develop short and medium-term plans to address gaps in reporting and strengthen capacity for timely annual reporting and analysis
2. Introduce training at pre-service level for health workers on the importance of and approach to assigning cause of death
3. Align overall and department specific annual hospital cause-specific mortality reporting with hospital level Integrated Disease Surveillance and Response and program specific morbidity and mortality reporting within annual reports
4. Strengthen existing Maternal and Perinatal Death Surveillance and Response efforts as a forerunner to extending the detailed case review approach to other medical disciplines with a focus on identifying lessons that improve quality and safety across the whole primary care system
5. Build analytical capacity at local and national levels as part of a strategic investment in clinical and population health epidemiology (and the institutions within which careers can develop) to enable greater data use and more sophisticated analyses as data from a wider array of population and health system indicators become available

## Abbreviations

DHIS: District Health Information System
IDSR: Integrated Disease Surveillance and Response
LMIC: Low and Middle Income Countries
OECD: Organisation for Economic Co-operation and Development
QI: Quality Improvement
SARS: Severe Acute Respiratory Syndrome
SDG: Sustainable Development Goals
